# Inclusion of Novel Olive Pulp: Impacts on Nutrient Digestibility, Rumen Fermentation, and Dairy Goat Performance

**DOI:** 10.3390/ani15213128

**Published:** 2025-10-29

**Authors:** Alberto Manuel Sánchez-García, Manuel Romero-Huelva, Noemí Pino-López, Isabel Jiménez-Romero, José Antonio Rosillo-Lozano, Antonio Ignacio Martín-García

**Affiliations:** 1 Estación Experimental del Zaidín, Consejo Superior de Investigaciones Científicas (CSIC), C/Prof. Albareda 1, 18008 Granada, Spain; alberto.sanchez@eez.csic.es (A.M.S.-G.); manuel.romero@eez.csic.es (M.R.-H.); noemi.p@eez.csic.es (N.P.-L.); isabel.jimenez@eez.csic.es (I.J.-R.); ecoturismodeguejarsierra@gmail.com (J.A.R.-L.); 2 Dcoop R&D Department, Ctra. Córdoba, 29200 Antequera, Spain

**Keywords:** dairy performance, digestibility, goats, novel olive pulp, olive by-products, ruminants

## Abstract

**Simple Summary:**

The use of agro-industrial by-products in animal feed is gaining relevance in the livestock sector. This study aimed to characterize and assess the effects of incorporating a novel olive pulp type into the diet of Murciano–Granadina goats, focusing on parameters such as ruminal fermentation, nutrient digestibility, energy and nitrogen metabolism, and milk production and quality. To achieve this, two in vivo trials were conducted to evaluate the impact of including olive pulp at a 12% inclusion rate in the concentrate. The results suggest that olive pulp can serve as an effective alternative feed ingredient for ruminants, offering promising potential for livestock nutrition.

**Abstract:**

In light of the exponential rise in feed costs within the livestock sector, the scientific research and valorization of novel agro-industrial by-products have essential strategies in animal nutrition. The overall objective of this study was to characterize and evaluate the inclusion of a novel olive pulp included at 12% of the concentrate on a dry matter basis in the diet of Murciano–Granadina goats to assess its effects on ruminal fermentation, nutrient digestibility, energy and nitrogen metabolism, and milk yield and composition. Two experiments were conducted, taking into account two groups (control group, CTL, and an experimental group) with the inclusion of 12% olive pulp in the concentrate (OPD): one in vivo trial in metabolic cages (n = 10 nulliparous female goats (34.1 ± 0.70 kg) per treatment) was conducted to evaluate digestibility, nitrogen balance, and energetic utilization; and a second on-farm production trial (n = 24 adult dairy goats (53.6 ± 1.14 kg) per treatment). The results showed no significant differences in energy balance or microbial protein synthesis between CTL and OPD (*p* > 0.05). However, the OPD exhibited higher digestibility of dry matter (71.2 vs. 68.8%; *p* = 0.028), organic matter (70.8 vs. 68.4%; *p* = 0.026), and crude fat (85.9 vs. 83.4%; *p* = 0.024), but lower crude protein digestibility (70.7 vs. 73.4%; *p* = 0.012) and nitrogen excretion (1.24 vs. 1.44 g/kg^0.75^; *p* < 0.001). Additionally, ruminal butyrate concentrations were higher in OPD goats (13.5 vs. 11.3 mol/100 mol of total short-chain fatty acids; *p* = 0.020). Although milk yield remained unaffected, the OPD exhibited higher milk protein (4.17 vs. 3.79%; *p* = 0.036) and conjugated linoleic acid (0.620 vs. 0.400%; *p* < 0.001) concentrations compared to CTL. These findings demonstrate that the inclusion of 12% of the novel olive pulp in goat concentrate is a viable feeding strategy that maintains productive performance while enhancing the nutritional quality of milk.

## 1. Introduction

The continuous growth of the global population is intensifying the need for innovative strategies to meet rising food demands. By 2050, the global population is projected to reach 10 billion people [1]. Given this projection and the current rate of resource consumption, the sustainability of food systems is increasingly at risk. In this context, the livestock sector is among the most affected, as climate change is affecting access to forage, the foundation of ruminant diets, by causing significant price increases, thus placing farmers in an economically vulnerable position.

One promising solution to this challenge is the integration of agro-industrial by-products (ABP) from the agri-food industry into livestock feeding systems. This approach offers potential benefits for both economic and environmental sustainability. Currently, approximately 16 million tonnes of ABP are generated annually in the European Union, with Spain being one of the leading producers, generating around 1.6 million tonnes each year [2].

The use of ABP in livestock feed has expanded globally, with ruminants being the primary beneficiaries due to their unique ability to extract energy from the fibrous components of these by-products [3]. Numerous studies have demonstrated that the inclusion of various fruit and vegetable by-products can positively influence ruminal fermentation, feed intake, and milk production in both small and large ruminants [4,5].

Among these by-products, olive by-products (OBP) are particularly relevant in Mediterranean regions, where olive oil production is widespread. Olive oil is one of the most widely consumed foods in the Iberian Peninsula, accounting for 95% of the total volume of oil produced, with an average consumption of 10.3 L per person per year in 2022 [6]. However, approximately 800 g of olive pomace are generated from every kilogram of processed olives [7]. Considering that olive oil production in Spain reached 765,300 tonnes during the 2023–2024 season, it is estimated that nearly one million tonnes of olive pomace were produced. This OBP, whose final reuse remains largely undefined, represents a significant environmental challenge due to its high organic matter (OM) content.

The inclusion of OBP in goat diets has been evaluated in several studies. Arco-Pérez et al. [4] reported no adverse effects on feed intake, nutrient digestibility, or milk production. In contrast, other studies, such as the one conducted by El Otmani et al. [8] have observed changes in milk composition of goats, including increased proportions of monounsaturated fatty acids (MUFA), polyunsaturated fatty acids (PUFA), and omega-9 fatty acids, particularly when OBP was included at levels up to 20% of the concentrate on a dry matter (DM) basis. The OBP exhibits an attractive nutritional profile, with a high DM content (approximately 90%), moderate crude protein (CP) levels (9–14%), and moderate crude fat (CF) content (4–6%), making it a suitable candidate for ruminant feeding strategies [9]. Some of the beneficial effects of OBP may be partially attributed to the bioactive compounds such as tyrosol, hydroxytyrosol, oleuropein, and verbascoside, which have demonstrated potent antioxidant and anti-inflammatory properties [10].

The continuous advances in olive oil extraction technologies have led to the development of different types of olive pomace, a solid by-product that can be classified based on its physicochemical properties, including moisture, residual oil content, and the proportion of remaining skin and pit fragments. One such variant, dry fatty pomace (commonly referred to as olive pulp, OP), is typically obtained through solvent-free extraction, followed by destoning and drying to a moisture content below 10%, with a residual fat content ranging from 5 to 10% on a DM basis. This novel fatty OP, also referred to by some authors as dried destoned virgin olive pomace (DDVOP), is recognized as a rich source of CP, beneficial fiber, phenolic compounds, tocopherols, oleic acid, and linoleic acid, making it a promising feed supplement for ruminant nutrition [11].

Sánchez-García et al. [12] evaluated the same OP as in the present study through in vitro cultures of ruminal microorganisms, comparing its fermentation profile with that of conventional feedstuffs and assessing different inclusion levels in a commercial concentrate. The lack of adverse effects on ruminal fermentation suggested that OP can be safely included up to 15% in ruminant diets. Likewise, Benincasa et al. [13] reported that DDVOP improved the degree of fatty acid unsaturation and increased the concentrations of phenolic compounds and tocopherols in sheep milk.

Previous studies have consistently shown that the inclusion of OP and other ABP, such as tomato or cucumber by-products, in dairy goats diets has consistently been associated with reductions in feed costs [7,14,15]. The market price of OP, which varies depending on energy costs, the value of alternative ingredients, and seasonal availability of OP from oil mills, is currently estimated at EUR 95/ton, which is substantially lower than that of other conventional ingredients used for ruminant diets (barley: EUR 192/ton; maize: EUR 222/ton [16]). These economic advantages highlight the potential of OP as a viable alternative ingredient in dairy goat diets. Beyond cost-effectiveness, its inclusion broadens the range of available feed resources and contributes to improving the overall economic efficiency of dairy goat production systems, aligning with sustainable feeding strategies that promote the valorization of ABP.

Based on this context, this study hypothesizes that the moderate CF content and the bioactive compounds present in OP may positively influence milk composition and, to a smaller degree, milk yield of dairy goats. Moreover, it is expected that inclusion of OP at 12 g DM per 100 g concentrate DM will not compromise nutrient digestibility, nitrogen (N) balance, energy utilization, or ruminal fermentation.

Therefore, the objective of this study was to characterize and evaluate the inclusion of a novel OP, derived from the olive oil industry, in the diet of dairy goats. Specifically, the study aimed to analyze the composition of OP and assess its effect on nutrient digestibility, energy balance, N utilization, ruminal fermentation parameters, milk production, and the fatty acid profile of the milk.

## 2. Materials and Methods

### 2.1. Animals

The management of the goats and the experimental procedures were carried out by qualified personnel in accordance with Royal Decree 53/2013, which regulates the protection of animals used in scientific research, and with European Directive 2010/63/EU. All procedures were reviewed and approved by the Animal Experimentation Ethics Committee of the Estación Experimental del Zaidín, evaluated by the CSIC Ethics Committee, and authorized by the Andalusian Government, the competent authority for animal welfare (Authorization 16/04/2021/047 dated 21 April 2021). All goats included in this study belonged to the Murciano–Granadina breed, selected for their high dairy aptitude and in accordance with the genetic improvement criteria established by the Spanish Association of Murciano–Granadina Breeders (Caprigran, Spain). A total of 68 goats were included, with 20 allocated to Experiment 1 and 48 to Experiment 2. Only female animals were used, as the study focused on evaluating the effects of OP inclusion on milk production. Environmental enrichment was provided in accordance with species-specific welfare standards and adapted to the housing conditions. In group housing systems, goats had free access to automatic scratchers, natural climbing structures (rocks, logs, and tires), and hiding areas. When individual housing was required for experimental purposes, pens were designed to allow visual and auditory contact with conspecifics, minimizing social isolation and promoting natural behaviors. Fresh water was continuously available via automatic waterers, and indoor thermal conditions were maintained using air conditioning systems to ensure animal comfort. Additionally, salt blocks were provided as mineral supplements and to stimulate exploratory and licking behaviors, supporting the expression of species-specific needs and enhancing overall welfare.

The pens were equipped with a mechanical underfloor slatted system for the efficient collection of feces and urine using automated scrapers, which prevented waste accumulation and ensured hygienic conditions. Air quality was continuously monitored and regulated through integrated ammonia and temperature sensors, maintaining optimal environmental parameters throughout the experimental period. Further details regarding the animals used in each experiment are provided in the corresponding sections.

### 2.2. Experiments

To address the objectives of this study, two sequential experiments were conducted, considering each animal as the experimental unit. First, an in vivo trial (Experiment 1) was performed to characterize the OP and evaluate the effect of its inclusion at 12 g per 100 g of concentrate DM on nutrient digestibility, N balance, and energy utilization in goats. This inclusion rate was determined according to the in vitro results reported by Sánchez-García et al. [12]. Subsequently, an on-farm trial (Experiment 2) was carried out to assess the impact of including OP at 12% DM in the concentrate of dairy goats on changes in live weight, milk yield, composition, and the fatty acid profile of milk in lactating goats.

### 2.3. Experimental Diets

The experimental diets for both trials were formulated based on findings from previous in vitro assays [12]. Accordingly, two experimental concentrates were prepared: a control concentrate (CTL), equivalent to a commercial dairy goat feed, and a treatment concentrate (OPC), containing 12% of OP on a DM basis. To complete the daily ration, oat hay was provided to achieve a forage-to-concentrate ratio of 30:70 (DM basis). The diets were formulated to meet the N and energy requirements of Murciano–Granadina goats for maintenance and production in Experiments 1 and 2, respectively [17]. Table 1 details the ingredients, chemical and mineral composition, and the cost of experimental concentrates, while Table 2 summarizes the fatty acid profile of the experimental diets.

### 2.4. Experimental Procedure

#### 2.4.1. Experiment 1: In Vivo Trial for the Evaluation of Nutrient Digestibility, N Utilization and Energy Balance from the Inclusion of OP in Goat Feed

The study involved 20 adults nulliparous female Murciano–Granadina goats (34.1 ± 0.70 kg), all aged 339 days and born on the same day. Animals were stratified by live weight and randomly assigned to two experimental groups (n = 10 goats per treatment). Group homogeneity was confirmed by one-way ANOVA, showing no significant differences in initial body weight (34.4 vs. 33.8 kg; *p* = 0.679). The goats were housed in individual pens with free access to water and fed twice daily (08:00 and 14:00) with a diet consisting of 50% concentrate and 50% oat hay. The only difference between groups was the type of concentrate: the control group (CTL) received a commercial concentrate, while the treatment group (OPD) received a concentrate formulated with 12% of OP on DM basis.

Before the experimental period, a 21-day adaptation phase was conducted, during which the animals were gradually acclimated to their new diets. On day 1, 20% of the previous concentrate was replaced by the new formulation. If no adverse effects were observed, this substitution was increased daily by 20% increments until complete replacement was achieved after five days. Throughout this adaptation period, individual feed intake was monitored to detect and address any notable differences in individual voluntary intake and forage-to-concentrate ratio.

Following the adaptation phase, a four-day trial was conducted to evaluate nutrient digestibility, energy, and N utilization, following the methodology described by Arco-Pérez et al. [4]. During this phase, goats were housed in metabolic cages with free access to water and were fed a diet with an average forage-to-concentrate ratio of 30:70 (DM basis). Daily measurements included concentrate and oat hay, feces, and urine. Feed and forage refusals were stored at 4 °C to preserve them until the subsequent chemical analysis. For feces, 20% of the daily output was collected and stored at –20 °C. Urine was collected in containers with 6 N HCl to maintain pH below 3 and prevent N volatilization. Of the total daily urine, 10% was pooled and stored at –20 °C, and an additional 2% was stored in 50 mL Falcon tubes for analysis of purine derivatives and creatinine.

On the final day of sampling, rumen fluid was collected at 10:00 am via esophageal intubation after an overnight fasting period. Samples were filtered through a double layer of sterile gauze and used to analyze ruminal fermentation parameters, including pH, short-chain fatty acids (SCFA), ammonia N (N-NH_3_), and lactic acid [18].

Animal body weights were recorded on the day prior to starting the adaptation period (day 0), during the adaptation period (day 12), and at the end of the trial (day 25) to monitor weight changes and assess potential treatment effects. Apparent nutrient digestibility was calculated based on nutrient intake and fecal output. For the assessment of energy and N utilization, losses through feces and urine were accounted for, while methane energy losses were estimated as 10.32% of digestible energy, following the methodology described by Aguilera et al. [19].

#### 2.4.2. Experiment 2: On-Farm Trial for the Evaluation of the Effect of the Inclusion of OP in the Diet of Dairy Goats on the Evolution of Live Weight, Milk Yield, Composition, and Fatty Acid Profile

A total of 48 adult lactating Murciano–Granadina dairy goats (53.6 ± 1.14 kg) with an average parity of 3.50 ± 0.255, were included in Experiment 2. The animals were managed under commercial farming conditions at “Las Viñas” farm (Guadix, Granada, Spain). Goats were stratified based on live weight, milk yield, parity, and days in milk, and then randomly assigned to two experimental groups (CTL and OPD; n = 24 goats per treatment). Group homogeneity was confirmed by one-way ANOVA, showing no significant differences between CTL and OPD groups (52.0 vs. 55.2 kg, *p* = 0.168; 2.16 vs. 1.91 kg milk/day, *p* = 0.247; 3.32 vs. 3.68 parity, *p* = 0.478; 265 vs. 274 days in milk (DIM), *p* = 0.603). The CTL group received a diet consisting of alfalfa hay and commercial concentrate in a 20:80 forage-to-concentrate ratio, while the OPD group was fed an equivalent diet, with the only difference being the inclusion of 12% of OP (DM basis) in the concentrate.

Throughout the 30-day trial, body weights were recorded on days 0 and 30. At the end of the experiment, both groups had reached 270 ± 8.19 DIM, for both CTL and OPD groups. On day 30, milk samples were collected for compositional analysis, including CF, CP, lactose, and somatic cell count. These analyses were performed using near-infrared spectroscopy with the CombiScope™ FTIR 600 Dairy Analyzer (Delta Instruments, Drachten, The Netherlands) at the Laboratory of Animal Production and Health of the Andalusian Regional Government (Junta de Andalucía, Spain). The fatty acid profiles of both milk and dietary fat were determined by gas chromatography at the Research, Technology, and Innovation Center of the University of Seville (Spain). Separation, identification, and quantification of fatty acid methyl esters were performed according to the methodology described by Arco-Pérez et al. [4]. The fatty acids were first extracted using the Folch method [20], and subsequently analyzed using a gas chromatograph (Agilent 6890N, Waldbronn, Germany) equipped with a flame ionization detector. The stationary phase consisted of an HP-88 capillary column (100 m length, 0.25 mm internal diameter, 0.20 µm film thickness). Hydrogen was employed as the carrier gas at a flow rate of 2 mL min^−1^. The analysis was conducted at the Agricultural Research Service of the University of Seville.

### 2.5. Sample Analysis, Calculations and Statistical Analysis

#### 2.5.1. Chemical Composition of the Diets

Chemical composition analyses of the diets were performed following the procedures described by the AOAC (2023) [21]. The determination of DM (method 934.01), OM (method 942.05), CF (method 920.39, via petroleum ether extraction), and N (method 984.13) was performed on feed offered, refusals, and feces. In the case of urine samples, only N content was determined. Neutral detergent fiber (NDF) and acid detergent fiber (ADF) were determined according to the method of Van Soest et al. (1991), using an ANKOM Fiber Analyzer (ANKOM Technology Corp., Macedon, NY, USA) [22]. Acid detergent lignin (ADL) was determined by solubilizing cellulose with 72% sulphuric acid. All fiber fractions were expressed on an ash-free basis. The energy content of the samples was determined using an adiabatic oxygen bomb calorimeter (Model 6100, Parr Instrument Company, Moline, IL, USA), which calculates the calorific value of the sample via direct calorimetry. Mineral content was analyzed by inductively coupled plasma optical emission spectrometry (ICP-OES) at the Scientific Instrumentation Service of the Estación Experimental del Zaidín, CSIC (Granada, Spain), following the procedure described by Pardo et al. [23].

#### 2.5.2. Determination of the Ruminal Fermentation Profile (SCFA and N-NH_3_)

The molar proportions of individual SCFA, including acetate, propionate, isobutyrate, butyrate, isovalerate, and valerate, were determined using a gas chromatography system coupled to a flame ionization detector (AutoSystem, PerkinElmer, Norwalk, CT, USA), following the procedure described by Arco-Pérez et al. [4]. Briefly, a cross-linked polyethylene glycol column was used, with crotonic acid as the internal standard. The SCFA identification was based on retention times relative to external standards, and quantification employed standard curves prepared in the same matrix as the rumen content samples. The N-NH_3_ concentrations were determined using colorimetric methods described by Weatherburn [24], which is based on the reaction of phenol, nitroprusside, and alkaline hypochlorite with N-NH_3_ to form indophenol blue. The resulting color intensity, measured spectrophotometrically at 690 nm, is directly proportional to the N-NH_3_ concentration in the sample.

#### 2.5.3. Purine Derivatives and Creatinine in Urine

The analysis of urine samples to determine creatinine and purine derivatives was performed at the Instrumentation Service of the Estación Experimental del Zaidín (CSIC, Granada, Spain) using the UPLC-ESI-QTof-MS technique. For this purpose, a UPLC Acquity H-Class liquid chromatograph (Waters, Milford, MA, USA) was coupled to a VION IMS QTof high-resolution ion mobility mass spectrometer (Waters, Milford, MA, USA). Before analysis, urine samples were centrifuged and filtered through 0.45 µm nylon filters and subsequently diluted with acetonitrile (1:1, *v*/*v*). Subsequently, a 200 µL aliquot of the diluted sample was mixed with 600 µL of a 1:1 (*v*/*v*) acetonitrile: water solution and stored at −20 °C until further analysis. Each sample was injected in duplicate for quantitative analysis.

#### 2.5.4. Calculations and Statistical Analysis

The CP content in the feed samples used across the experiments was calculated by multiplying the N concentration by a factor of 6.25. The proportion of non-fiber carbohydrates (NFC) was estimated using the following formula: NFC (%) = 100 − (Ash + CP + CF + NDF). For the economic estimation of the concentrate cost, a price comparison was performed based on the ingredient composition of both diets, including only those components whose contribution exceeded 1%. For the price of OP, the 2025 market value was considered, which was EUR 95/ton. Metabolic weight was calculated using the formula: Metabolic weight (kg^0·75^) = live weight^0·75^. The urinary concentrations of purine derivatives, initially expressed in mg/L from the analytical measurements, were converted to μM using the molecular weights of each compound: hypoxanthine (136.1 g/mol), allantoin (158.1 g/mol), xanthine (152.1 g/mol), uric acid (168.1 g/mol), and creatine (113.1 g/mol).

Daily production of CF, lactose, and CP in milk was calculated by multiplying the daily milk yield by the percentage of each respective component. Specifically, CF, lactose, or CP production (g/d) = daily milk yield (g/d) × % CF, lactose, or CP, respectively. Total milk solids were calculated as the sum of the percentages of CF, CP, and lactose present in the milk. Additionally, the fat-protein corrected milk (FPCM) was calculated taking into account the formula proposed by Mancilla-Leytón et al. [25]: FPCM = Milk yield (g/day) × [(0.10 × CF + 0.08 × CP + 0.20)], which is specific for Murciano–Granadina goats based on average milk CF and CP values reported by Marcos et al. [26].

The atherogenicity index of milk was calculated using the ratio: SFA/(MUFA + PUFA). The omega-6/omega-3 ratio was calculated by dividing the sum of omega-6 fatty acids (C18:2n6, C18:3n6, C20:2n6, C20:3n6, C20:4n6) by the sum of omega-3 fatty acids (C18:3n3, C20:5n3, C22:5n3, C22:6n3).

The apparent digestibility of nutrients (%) was calculated as (1 − fecal output/intake) × 100. The following formulas were used for the analysis of energy and N metabolism:

- Digestible N (g/kg^0.75^) = (N intake − N in feces)/Metabolic weight.
- N balance (g/kg^0.75^) = (Digestible N − N in urine)/Metabolic weight.
- Digestible energy (DE) (MJ/kg^0.75^) = (Energy intake − Energy in feces)/Metabolic weight.
- Metabolizable energy (ME) (MJ/kg^0.75^) = (DE − Energy in urine − Methane energy)/Metabolic weight.

The statistical analyses were performed using ANOVA with the SPSS software package (version 29.0.0.0; IBM Corp., Armonk, NY, USA), considering diet type as the main fixed factor. Sample size was calculated using G-Power software (version 3.1.9.6) with the following parameters: Cohen’s d = 0.8, α = 0.05, power = 0.80, and 1:1 allocation between CTL and treatment groups. Expected variability was estimated from previous experiments, selecting the parameter with the most relevant standard deviation (total SCFA and milk yield, respectively, for Experiments 1 and 2) to determine the minimum number of animals per group. This approach ensured the detection of biologically relevant differences while minimizing Type I and Type II errors and adhering to ethical reduction principles. To minimize potential confounding, treatment order was always randomized, and sampling in Experiment 1 was alternated between animals from each treatment. Data normality was assessed using the Shapiro–Wilk test. When normality assumptions were not met, appropriate data transformations or alternative non-parametric tests were applied to ensure the validity of the statistical analyses. For the analysis of purine derivatives in urine, a univariate ANOVA was conducted, including creatinine concentration as a covariate to adjust for individual variations in urine dilution. Statistical significance was set at *p* ≤ 0.05, while values between 0.05 < *p* < 0.10 were interpreted as trends. Effect sizes were evaluated using Cohen’s *d* to provide additional insight into the magnitude of the observed differences. For consistency, all results are presented with the OPD group first, followed by the CTL group.

## 3. Results

### 3.1. Experiment 1: In Vivo Trial for the Evaluation of Nutrient Digestibility, N Utilization and Energy Balance from the Inclusion of OP in Goat Feed

These results provide an approximate characterization of the experimental feeds used in the nutrient digestibility trial. Based on the nutritional composition of the experimental concentrates (CTL and OPC) used in both the digestibility and on-farm milk production trials (Table 1), both concentrates exhibited similar overall nutritional and mineral profiles. However, the CTL concentrate contained approximately 16% more NDF and ADF compared to the OPC, while ADL content was 8% lower than that of the OPC. The CP content was 13% higher in the CTL concentrate, whereas the CF content did not differ between treatments. The mineral composition analysis revealed that OP is particularly rich in K (3.15 g/100 g) and Mg (0.40 g/100 g), in comparison with oat hay and both experimental concentrates.

Based on the ingredient composition of the concentrates and considering only components contributing more than 1%, it was determined that the OPC was more economical, with a cost difference of EUR 17.9/ton compared to the CTL concentrate.

The fatty acid composition analysis of OP, oat hay, and the CTL and OPD concentrates revealed that C18:1n9c (oleic acid) was predominant in OP, with a concentration of 50.4%, which was reflected in its high MUFA content (56.3%). Furthermore, the MUFA and PUFA proportions were slightly higher in the OPC compared to CTL concentrate. Oat hay was characterized by a high SFA content (52.9%), approximately 22% greater than in both concentrates and OP. The repeated occurrence of C16:1 in the dataset results from the detection of two structurally distinct isomers of this fatty acid. The concentrations of other fatty acids not included in Table 2 were likely below the detection limit.

Following the experimental phase, one animal per group was excluded due to erratic feed intake, resulting in anomalous data. Based on the results obtained for the apparent digestibility trial (Table 3), a significant increase in the DM (71.2 vs. 68.8%; *p* = 0.028), OM (70.8 vs. 68.4%; *p* = 0.026), and CF (85.9 vs. 83.4%; *p* = 0.024) digestibility was observed in the OPD treatment compared to the CTL. Conversely, the CTL exhibited a higher apparent digestibility of CP (707 vs. 734 g/kg; *p* = 0.012). Finally, according to fiber digestibility, no significant differences were found between the treatments evaluated (NDFD: *p* = 0.876; ADFD: *p* = 0.985).

The N utilization data indicated that N intake (1.57 vs. 1.89 g/kg^0.75^; *p* < 0.001), N excretion (1.24 vs. 1.44 g/kg^0.75^; *p* < 0.001), and N balance (0.326 vs. 0.487 g/kg^0.75^; *p* = 0.042) were significantly lower in the OPD group compared to CTL. This difference in N excretion was evident in both urine and feces. Additionally, digestible N (1.11 vs. 1.39 g/kg^0.75^; *p* < 0.001) and the ratio of digestible N to N intake (70.7 vs. 73.4%; *p* = 0.012) were significantly lower in the OPD compared to the CTL group. However, when creatinine was included as a covariate to adjust for renal function, no significant differences were observed between treatments in the excretion of purine derivatives (allantoin: *p* = 0.684; hypoxanthine: *p* = 0.347; xanthine: *p* = 0.515; uric acid: *p* = 0.613).

Regarding energy balance, significant differences were observed only in fecal energy losses (0.313 vs. 0.352 MJ/kg^0.75^; *p* = 0.018), which were slightly higher in the CTL group. Additionally, there was a trend toward a higher proportion of DE relative to GEI (69.2 vs. 66.7%; *p* = 0.094) in the OPD group compared to CTL. No significant differences were observed in the estimated CH_4_ production (MJ/day) between treatments (*p* = 0.696).

Considering the ruminal fermentation parameters (Table 4), based on rumen contents collected via esophageal intubation following the digestibility trial conducted in metabolic cages, a significant difference was observed in the proportion of butyrate, which was higher (*p* = 0.020) in the OPD group compared to CTL. In contrast, N-NH_3_ concentrations and the remaining SCFA concentrations did not differ significantly between diets.

### 3.2. Experiment 2: On-Farm Trial to Evaluate the Effects of the Inclusion of OP in Dairy Goat Feeding: Animal Performance, Milk Yield, Composition and Fat Lipid Profile

#### 3.2.1. Animal Performance

A total of 41 adult female Murciano–Granadina goats (n = 17 and n = 24 goats for CTL and OPD, respectively) were included in the final data analysis. Initially, 48 animals were assigned to the study; however, 7 goats from the control group (CTL) were excluded. Of these, 3 animals failed to conceive during the breeding period, and 4 were considered missing values for body weight measurements. These exclusions were necessary to ensure the reliability and consistency of the experimental data. Regarding changes in body weight before and after the trial, the CTL group showed a weight gain of 2.70 kg/per goat, while the OPD group gained 2.60 kg per goat. However, there were no significant differences in pre-trial (*p* = 0.168) or post-trial (*p* = 0.192) weights between groups (Table 5).

#### 3.2.2. Milk Yield, Composition and Fatty Acid Profile

For the analysis of lactational performance, a total of 38 animals were included (n = 18 and n = 20 goats for CTL and OPD, respectively). Although 48 goats were initially enrolled in the study and 40 were intended to be used for the analysis of milk yield, composition, and fatty acid profile analyses, two goats from the CTL group were excluded from the statistical analysis of milk yield and composition due to missing values detected during milk recording. These exclusions were made to ensure the accuracy and reliability of the dataset. After 30 days of consuming the experimental diets (Table 6), milk yield was not significantly affected by diet, with average daily productions of 2160 and 1920 g of milk/day for CTL and OPD, respectively (*p* = 0.269). Similarly, no significant differences were observed in FPCM (2282 vs. 2113 g/day; *p* = 0.411) between the two experimental groups.

No significant differences in CF concentration between treatments (5.86 vs. 5.65%; *p* = 0.465). However, the OPD group exhibited a significantly higher CP concentration (4.17 vs. 3.79%; *p* = 0.036) and a lower lactose content (4.58 vs. 4.76%; *p* = 0.008) compared to the CTL group. According to the ABTS assay, which evaluates the antioxidant activity of milk, no significant differences were found between groups (*p* = 0.540).

Regarding the fatty acid composition of the milk (Table 7), significant differences were observed between treatments for several components. Among essential fatty acids, the OPD group showed a lower proportion of arachidonic acid (C20:4n6; 0.228 vs. 0.271%; *p* = 0.035) compared to CTL group. Conversely, the OPD group showed a significantly greater concentration of alpha-linolenic acid (C18:3n3 α; 0.552 vs. 0.239%; *p* < 0.001) relative to the CTL.

Additionally, the PUFA/MUFA ratio was significantly higher in the OPD compared to the CTL group (0.210 vs. 0.190; *p* = 0.031). No significant differences were observed in the proportion of trans fatty acids between treatments (*p* = 0.733). The omega-6/omega 3 fatty acid ratio was significantly lower in the OPD group compared to the CTL (4.00 vs. 6.19; *p* < 0.001). Furthermore, the concentrations of odd-chain saturated fatty acids C15:0 (1.01 vs. 0.699%; *p* < 0.001) and C17:0 (0.839 vs. 0.405%; *p* < 0.001) were significantly greater in the OPD group. Lastly, the content of conjugated linoleic acids (CLA) was also significantly higher in the OPD group compared to the CTL (0.62 vs. 0.40%; *p* < 0.001).

## 4. Discussion

The by-products generated by the olive oil industry represent a potentially valuable resource that could be repurposed to enhance ruminant nutrition. Recent advances in olive oil extraction technologies have led to the development of a novel type of OP, whose nutritional potential and effects on animal performance remain largely unexplored. The main objectives of this study were to characterize the chemical composition of this novel OP and to evaluate its effects on the metabolic parameters and lactational performance of dairy goats.

### 4.1. Chemical Composition of OP

Analyzing the chemical composition of the OP and the experimental concentrates is essential to understanding the nutritional and mineral contributions, as well as identifying key differences between diets.

The chemical composition of the OP used in this study was comparable to those reported by Marcos et al. [27] and Molina-Alcaide and Yáñez-Ruíz [15] for olive cake, a by-product typically obtained from secondary extraction processes aimed at recovering residual oil from olive. Notably, the OP analyzed here showed higher CF and GE contents, and lower fiber fractions (NDF, ADF, ADL), which may be attributed to its mechanical defatting process without the use of chemical solvents. In contrast, the olive cake is commonly subjected to solvent extraction (e.g., hexane) to remove fat, which leads to a reduction in CF and GE levels. Regarding CP content, our results are consistent with those reported by Marcos et al. [27]. Furthermore, the OP used in our study underwent a drying process that reduced its moisture content less than 10%, thereby increasing its DM content. Overall, the OP used in this study can be characterized as a more energy-dense and less fibrous ingredient compared to conventional olive cake, making it suitable for inclusion in ruminant diets and potentially enhancing overall diet digestibility and animal performance.

Molina-Alcaide et al. [28] conducted a nutritional evaluation of different types of OBP, including OP obtained from an experimental olive oil extraction process involving the complete removal of the olive stone prior to milling. This OP was analyzed both in its raw form and after near-complete oil extraction. When comparing the chemical composition of the OP used in the present study with the values reported by Molina-Alcaide et al. [28], it can be observed that most nutrient concentrations fall between those of the intact and the defatted OP described by those authors. Notably, the OP in the present study shows a substantially higher CP content (34.0% more) and a lower CF content (8.35% less) compared to the unextracted OP reported by Molina-Alcaide et al. [28]. Additionally, the proportion of cell wall components in our OP was 9.90% higher than in their unextracted sample. These differences may primarily reflect inefficiencies in the stone removal process applied in our study, potentially resulting in a greater inclusion of fibrous material associated with residual stone fragments.

Regarding trace minerals, our results consistently showed higher concentrations (averaging 77% greater) than those reported by Hassan et al. [29] for conventional non-dehulled and extracted olive cake. The elevated Cu content observed in OP is primarily attributed to the application of Cu-based fungicides commonly used in olive groves to manage fungal pathogens. Notably, in vivo studies conducted by Yáñez-Ruíz and Molina-Alcaide [30] demonstrated that sheep are more sensitive than goats to dietary Cu levels exceeding 60 mg/kg DM. This species-specific difference suggests that, although caution may be warranted when incorporating OBP into sheep diets, the Cu concentrations detected in OP are unlikely to pose a risk for dairy goats. This is further supported by the findings of Arco-Pérez et al. [31], who reported that the inclusion of olive pomace in dairy goat diets did not cause significant alterations in trace mineral levels in plasma, urine, or milk, thus confirming the safety of OP use with respect to trace mineral balance in this species.

### 4.2. Digestibility Assay, N Utilization and Energy Balance

In the nutrient digestibility experiment, concentrate intake was comparable between groups, indicating that the inclusion of OP at 12% DM in the diet did not negatively affect feed acceptability. These findings align with the review by Correddu et al. [32], which reported that the inclusion of ABP generally did not reduce voluntary feed intake in goats, although it tended to decrease intake in sheep. Such differences may be explained by the fact that goats have developed adaptive strategies to mitigate the impact of polyphenols on feed intake. Although the animals were initially offered a 50:50 forage-to-concentrate ratio during the adaptation period, voluntary intake shifted toward an average ratio of 30:70, with the OPD showing a relatively higher concentrate proportion (27:73) compared to the CTL group (30:60). These results are consistent with the findings of Arco-Pérez et al. [4], who reported no significant differences in the intake of a total mixed ration containing olive cake and a CTL diet in dairy goats of the same breed used in the present study.

The slight differences observed in both the forage-to-concentrate ratio and the nutritional composition of the two concentrates may explain the variations in nutrient intake between treatments. Our findings contrast with those of Obeidat and Thomas [33], who reported no significant differences in nutrient intake in male black goat kids, except for CF intake, which increased when olive cake was included at levels of 7.5% and 15% compared to a CTL diet. It is important to highlight that in their study, the CF content was higher when olive cake was included, whereas in our study, the inclusion of OP did not result in differences in CF content between the dietary treatments.

The significantly increased digestibility of DM, OM, and CF observed in the OPD group, despite no differences in intake, aligns with and complements findings from several previous studies. For instance, Obeidat and Thomas [33] reported a higher CF digestibility coefficient when olive cake was included at 15% of dietary DM, similar to the enhancement in the CF digestibility seen in our OPD treatment. Likewise, Arco-Pérez et al. [4] observed improvements primarily in CF digestibility with the inclusion of OBP in dairy goats’ diet, consistent with our results.

The increase in OM digestibility may be consistent with the in vitro findings of Sánchez-García et al. [12], who reported a similar enhancement in OM digestibility when the same novel OP was at levels up to 15% of the concentrate. It is important to highlight, however, that in the in vitro context, these effects were not statistically significant, a discrepancy likely due to the absence of complex physiological and metabolic processes such as ruminal microbiome dynamics, gastrointestinal motility, and animal metabolic processes that are inherently present in vivo and may significantly impact nutrient utilization.

The greater DM digestibility observed in the OPD group may be partially explained by the higher NFC content (22.7% greater) in the OPC compared to the CTL. This additional fermentable substrate likely enhanced microbial activity and nutrient breakdown. Conversely, the 4% reduction in CP digestibility in the OPD treatment could be attributed to the elevated ADL content, as well as the presence of phenolic compounds known to form indigestible complexes with proteins, thereby limiting their availability.

Regarding N utilization, the higher N intake and excretion in the CTL group compared to the OPD group have important environmental implications. The reduced N excretion observed with the OPD diet may help lower N_2_O emissions from ruminant production systems. Nitrous oxide is a greenhouse gas with a global warming potential approximately 298 times greater than that of CO_2_ over 100 years [34]. The N utilization values in our study are consistent with those reported by Arco-Pérez et al. [4], who observed lower Digestible N/intake N and N balance/digestible N associated with the diets that included OBP silages. The decreases in digestible N and N balance associated with the OPD diet may be influenced by the presence of compounds such as ADL and secondary plant metabolites, such as tannins. These substances may promote the formation of protein-fiber complexes, which are less digestible and therefore reduce the availability and utilization of N in the animal. Supporting this mechanism, Martín-García et al. [35] reported that disrupting such complexes in OBP using polyethylene glycol notably enhanced N availability in vitro.

In terms of energy balance, the increase in fecal energy in the CTL group may be attributed to the greater fecal N excretion and higher fiber intake, despite no changes in fiber digestibility coefficients. The elevated fiber passage likely resulted in greater energy losses in feces, thus lowering the overall energy efficiency compared to the OPD group. Finally, no significant differences were observed in purine derivatives concentrations between groups, which is related to the microbial protein synthesis; however, a reduction in N utilization was detected in the OPD group. This apparent discrepancy may be explained by a lower post-ruminal N metabolism efficiency or reduced CP digestibility, as previously reported in studies evaluating the effects of polyphenol-rich plant extracts on rumen fermentation [36]. In that study, despite no changes in fermentation profiles, the presence of hydrolyzable tannins and flavonoids, which could be present in the OP, was associated with reduced N-NH_3_ and branched-chain fatty acid production, suggesting a shift in N partitioning rather than microbial protein synthesis.

### 4.3. Ruminal Fermentation Parameters

Regarding the ruminal fermentation parameters, the observed increase in butyrate concentration could be particularly noteworthy from a milk production perspective, as a higher proportion of this SCFA could enhance de novo fatty acid synthesis in the mammary gland. This effect could potentially lead to increased milk yield and higher CF content, thereby improving cheese extract [37]. However, our results did not show this effect in the on-farm performance trial. It is important to emphasize that although significant differences were detected in the in vivo digestibility trial, such differences may not necessarily be replicated in the context of the on-farm trial, especially since SCFA concentrations were not measured. In line with previous studies, where de-stoned OP was included at 5% and 9% of the diet (DM basis), a significant increase was observed in the concentrations of acetate, propionate, butyrate, and isovalerate, as well as in the acetate-to-propionate ratio (A:P) [38]. Our results align with these findings regarding butyrate concentration; however, the other SCFA did not differ significantly between treatments. This discrepancy might be attributable to the lower inclusion rate in the referenced studies compared to our current trial, as well as possible variations in experimental design and analytical methods.

### 4.4. Milk Production, Crude Composition and Fatty Acid Profile

The findings related to milk production and composition are consistent with those reported by Arco Pérez et al. [4], who found that the inclusion of OBP silage in the diet of Murciano–Granadina goats did not affect milk yield and the CF concentration. However, in the present study, milk composition showed a higher CP and lower lactose concentration in the OPD group. This result was unexpected and contrasts with the findings of Keles et al. [39], who reported that the inclusion of stoned olive cake silage did not alter milk composition in dairy goats. Conversely, our findings are supported by Castellani et al. [40] and Marcos et al. [26], who observed a significant increase in milk CP concentration in dairy cows and goats fed diets including OP, where defatted olive cake was included at 10% and 8% of the diet on a DM basis, respectively. The increase in milk CP content may be attributed to tannins present in OP, which could enhance post-ruminal N availability by protecting dietary CP from ruminal degradation, thereby improving amino acid absorption in the small intestine [41].

The higher concentrations of odd-chain fatty acids C15:0 and C17:0 observed in the milk of the OPD group are noteworthy due to their association with a reduced risk of type 2 diabetes and cardiovascular disease in human consumers [42,43,44]. These fatty acids are synthesized in the rumen through the elongation of propionate and valerate, and are positively correlated with propionate production, thereby reflecting potential shifts in ruminal fermentation patterns [45]. However, the in vivo digestibility trial conducted in this study did not show significant differences in the proportions of propionate or other SCFAs between treatments. This discrepancy suggests that the differences in ruminal fermentation leading to increased odd-chain fatty acid synthesis may be more pronounced under on-farm conditions, which can differ substantially from controlled experimental settings. Additionally, an increase in C17:0 and C17:1 fatty acids, along with a decrease in C14:0, as reported in other studies, though not in the present study, has been proposed as a biomarker for early detection of subacute ruminal acidosis before clinical symptoms appear [46].

Interestingly, the OPD group exhibited a notable increase in CLA concentration. These types of PUFA have numerous beneficial effects on human health, including anti-obesity, anticarcinogenic, antihypertensive, and antidiabetogenic properties, among others, with dairy products being a primary dietary source [47]. Beyond human health benefits, specific CLA isomers may also protect mammary epithelial cells from lipid peroxidation, offering potential benefits for the animal itself [48]. The CLA content in the OPD group reached 6.20 mg/g of CF, compared to 4.00 mg/g in the CTL group. This increase may be attributed to the higher polyphenol content in the OPC, which could modulate the ruminal biohydrogenation of unsaturated fatty acids [30]. As a result of this modulation, the complete hydrogenation of vaccenic acid (VA) to stearic acid in the rumen is reduced, allowing a greater amount of VA to reach the mammary gland [46]. There, VA serves as a substrate for CLA synthesis, ultimately increasing its concentration in milk [49]. Our findings align with those of Molina-Alcaide et al. [50] and Castellani et al. [40], who reported an increase in CLA levels in milk from goats and cows fed with diets containing OBP at an inclusion rate of 10%, respectively.

Regarding trans fatty acids, no significant differences were observed between treatments, with values remaining at the lower end of the range reported in other studies [51]. This finding is favorable from a human health perspective, given the well-established link between trans fats and increased risk of coronary heart disease and other adverse health outcomes [52].

By analyzing the antioxidant composition of milk obtained from both groups, the absence of differences in the ABTS test was unexpected. Theoretically, OP inclusion should elevate antioxidant concentrations both systemically in the animal and in the milk, as demonstrated by Ianni et al. [53]. Supporting this, Benincassa et al. [13] reported that ewe diets supplemented with 6% DDVOP increased the proportions of PUFA and MUFA in milk, concomitant with elevated phenol and tocopherol content. These bioactive compounds contribute antioxidant properties that could enhance milk quality. Similarly, an increase in total phenolic content in milk would be expected with the inclusion of OP, as reported by Litrenta et al. [54], who found that dietary supplementation with olive cake at 10% and 20% significantly elevated total phenols in cow’s milk compared to a CTL.

However, the transfer of dietary antioxidants such as polyphenols to milk is a complex process, often limited by extensive metabolic transformations occurring within the rumen and the animal’s systemic metabolism. Polyphenols can be extensively degraded by ruminal microbes or undergo conjugation and subsequent excretion, thereby reducing their bioavailability for mammary secretion [55]. Supporting this complexity, Valenti et al. [56] reported lower total phenolic concentrations in the milk of sheep fed pomegranate pulp, a by-product rich in antioxidant compounds, likely due to the higher PUFA content in milk. These PUFAs are highly susceptible to oxidation and may influence the overall antioxidant profile detected, potentially masking the presence of phenolic compounds.

### 4.5. Suitability of the Use of OP for the Dairy Goat Sector

This study demonstrates that incorporating OP into pelleted concentrate formulations at a 12% of DM for dairy goats could be considered a significant advancement over previous approaches, such as the silage-based use of OBP described by Arco-Pérez Et Al. [4]. The inclusion of OP in dry concentrate formulations offers distinct practical advantages, including reduced contamination risk, greater handling efficiency, improved feed acceptability, especially in the case of goats, and extended shelf life. Furthermore, this work expands the current understanding of ABP revalorization in ruminant nutrition.

Despite our results supporting the suitability of OP for dairy goat feeding, several limitations of this work should be noted. Firstly, this study lacks a comprehensive economic assessment, mainly due to the fluctuating prices of raw materials, especially OP, which is highly influenced by the volatility of olive oil prices. Nevertheless, based on the available data during this study (when olive oil prices were particularly high due to the drought), we estimate that the OPC feed could represent a cost saving of approximately 2.32% for farmers compared to the CTL feed. Considering the current OP prices in 2025, which are close to EUR 95/ton, the savings could reach up to 7.84% with the use of OPC feed compared to the CTL feed. On the other hand, the potential reduction in environmental impact derived from the incorporation of OP in the dairy goat diet was not assessed. Therefore, it is necessary to determine this impact objectively and realistically, using tools such as the carbon footprint of goat milk or a full life-cycle assessment (LCA), following the strategy outlined in recent approaches [57]. Finally, given the complexity of the ruminant digestive system (microbiologically, physiologically, and anatomically) and its usual requirement for adaptation periods of varying length to dietary changes, a longer observation period covering the entire lactation cycle, including late lactation, is needed to fully understand the effects on N utilization.

## 5. Conclusions

This study successfully characterized OP as a suitable by-product for dairy goat nutrition. The inclusion of OP at 12% in concentrate, on a DM basis, preserved nutrient digestibility, energy balance, and N utilization efficiency without adverse effects on ruminal fermentation parameters. Milk yield remained unaffected, while milk composition was enhanced through increased CP content and an improved fatty acid profile, particularly CLA. The partial substitution of conventional ingredients with OP reduced concentrate costs, demonstrating its economic viability. While these findings validate OP as an effective feed ingredient that meets the study objectives of characterizing and evaluating its dietary inclusion effects, further research is needed to quantify long-term economic impacts and environmental benefits through LCA.

## Figures and Tables

**Table 1:** Ingredients, chemical and mineral composition, and feed cost of experimental dietary components used in Experiments 1 and 2.

			Concentrates	
Ingredients (% DM)	OP	Oat Hay	CTL	OPC
Bran			17.0	15.0
Maize grain			20.0	17.6
Soya (47% CP)			19.6	17.3
OP			-	12.0
Soybean hull			11.4	10.0
Maize DDGS			6.42	5.65
Sugarbeet pulp			9.96	8.77
Barley			4.55	4.00
Wheat			7.48	6.59
Additives ^1^			3.61	3.18
Total			100	100
Nutrients (% DM)				
DM	91.5	86.1	88.4	89.2
OM	88.2	81.0	93.2	93.4
NFC	21.2	12.7	32.9	40.5
CP	9.87	14.4	19.5	17.2
CF	7.90	2.40	4.03	4.03
NDF	49.2	51.5	36.7	31.7
ADF	35.9	26.9	17.3	15.0
ADL	24.5	5.01	4.98	5.37
GE (MJ/kg DM)	21.0	15.8	17.9	18.6
Minerals (%)				
Ca	1.05	2.44	0.75	0.71
K	3.15	1.54	0.94	0.98
Mg	0.40	0.21	0.32	0.22
Na	0.14	0.53	0.48	0.31
P	0.27	0.20	0.47	0.38
S	0.14	0.29	0.26	0.20
Essential trace minerals (mg/kg)				
Al	1012	2032	182	205
Cr	16.4	5.33	2.85	2.62
Cu	26.7	24.7	8.55	7.79
Fe	718	1276	282	237
Mn	56.8	100	143	91.8
Ni	12.8	4.19	2.95	2.81
Si	533	98.1	347	337
V	0.93	2.69	0.01	<0.01
Zn	30.9	49.2	179	129
Non-essential trace minerals (mg/kg)				
Sr	48.0	185	22.0	23.9
Ti	2.39	3.73	1.61	1.64
Concentrate cost (EUR/ton)			246.8	228.9

OP: Olive pulp; CTL: control concentrate; OPC: concentrate containing 12% of OP; DM: dry matter; OM: organic matter; NFC: non-fiber carbohydrates; CP: crude protein; CF: crude fat; NDF: neutral detergent fiber; ADF: acid detergent fiber; ADL: acid detergent lignin; GE: gross energy; DDGS: distiller’s dried grains with solubles; ^1^ Additives: soybean oil (CTL = 1.20%, OPC = 1.06%), NaHCO_3_ (1.00%), mineral mixture (0.34%: Ca 18.0%, P 9.0%, Mg 4.0%, Na 12.0%, K 1.0%, Cu 0.12%, Zn 0.55%, Mn 0.15%, Se 0.003%, Co 0.01% and I 0.025%), CaCO_3_ (0.33%), salt (0.23%), Buffering agent (0.20%), Ca(H_2_PO_4_)_2_ (0.10%), mycotoxin adsorbent Mycofix Plus 3.E^®^ (0.10%), probiotics Actisaf SC47 HR+^®^ (0.08%), and flavoring agent Luctarom 2503Z^®^ (0.03%).

**Table 2:** Fatty acid composition of olive pulp, oat hay and experimental concentrates (CTL and OPC).

			Concentrates	
Fatty Acids (g/100 g)	OP	Oat Hay	CTL	OPC
C8	0.063	0.144	-	0.066
C10	0.778	1.41	0.237	0.344
C11	-	-	0.142	0.176
C12	-	1.05	0.582	0.142
C14	0.282	2.50	0.992	0.466
C14:1	2.19	-	-	0.500
C15	0.236	-	-	-
C15:1	-	0.287	-	-
C16	16.2	29.9	23.4	18.6
C16:1	1.27	1.00	0.254	0.452
C16:1	2.22	8.80	1.67	2.51
C17	4.10	-	-	0.950
C17:1	0.224	-	-	-
C18	6.68	18.0	5.53	7.29
C18:1n9c	50.4	8.86	24.9	24.4
C18:2n6c	12.0	12.3	37.8	39.6
C18:3n6 γ	0.051	-	-	-
C18:3n3 α	1.08	14.5	3.44	3.87
C20	0.535	-	-	-
C20:2	0.241	1.06	0.975	0.304
C21	1.29	-	-	0.306
C20:3n3	0.172	0.254	0.090	0.076
Summary				
SFA	30.1	52.9	30.9	28.3
MUFA	56.3	18.9	26.8	27.9
PUFA	13.6	28.1	42.3	43.9

OP: olive pulp; CTL: control concentrate; OPC: concentrate containing 12% of OP; SFA: saturated fatty acids; MUFA: monounsaturated fatty acids; PUFA: polyunsaturated fatty acids.

**Table 3:** Effect of olive pulp inclusion on metabolic weight, nutrient intake, apparent digestibility, N and energy balance and urinary excretion of creatine and purine derivatives in goats (n = 9 goats per treatment).

	Diets			
	CTL	OPD	SEM	*p*-Value
Metabolic weight (kg^0.75^)	14.1	14.2	0.213	0.814
Intake (g DM/day)				
Oat hay	270	227	17.3	0.224
Concentrate	619	619	2.70	0.972
Intake (g DM/kg^0.75^)	63.4	59.7	1.05	0.084
Nutrient intake (g/day)				
DM	889	846	17.7	0.235
OM	770	735	15.6	0.274
CP	166	139	4.41	<0.001
CF	30.3	28.7	0.497	0.115
NDF	349	297	11.4	0.016
ADF	180	157	5.24	0.025
GE (MJ/day)	14.8	14.4	0.306	0.517
Apparent digestibility (%)				
DMD	68.8	71.2	0.545	0.028
OMD	68.4	70.8	0.550	0.026
CPD	73.4	70.7	0.555	0.012
NDFD	51.4	50.9	1.32	0.876
ADFD	49.2	49.3	1.05	0.985
CFD	83.4	85.9	0.599	0.024
N utilization (g/kg^0.75^)				
N intake	1.89	1.57	0.049	<0.001
N excretion	1.44	1.24	0.033	<0.001
N balance ^1^	0.487	0.326	0.031	0.042
Urine N	0.939	0.781	0.028	0.001
Fecal N	0.503	0.458	0.011	0.066
Digestible N ^2^	1.39	1.11	0.041	<0.001
Digestible N/N intake (%)	73.4	70.7	0.557	0.012
N balance/Digestible N (%)	32.2	29.0	1.88	0.418
Energy balance (MJ/kg^0.75^)				
GEI	1.06	1.02	0.017	0.161
Fecal energy	0.352	0.313	0.009	0.018
Urine energy	0.041	0.040	0.001	0.986
DE ^3^	0.704	0.704	0.013	0.696
Methane energy ^5^	0.073	0.073	0.001	0.696
ME ^4^	0.591	0.592	0.011	0.692
DE/GEI (%)	66.7	69.2	0.627	0.094
ME/GEI (%)	55.9	58.1	0.559	0.157
Creatine and purine derivatives in urine (µM)				
Creatine	4736	4298	207	0.304
Allantoin	6380	6162	256	0.684
Hipoxanthine	642	568	38.1	0.347
Xanthine	18.4	16.6	1.30	0.515
Uric acid	397	346	47.7	0.613
Total	7437	7093	297	0.578

CTL: control group; OPD: group including 12% OP in the concentrate; SEM: standard error of mean; DM: dry matter; OM: organic matter; CP: crude protein; CF: crude fat; NDF: neutral detergent fiber; ADF: acid detergent fiber; GE: gross energy; DMD: dry matter digestibility; OMD: organic matter digestibility; CPD: crude protein digestibility; NDFD: neutral detergent fiber digestibility; ADFD: acid detergent fiber digestibility; CFD: crude fat digestibility; ME: metabolizable energy; GEI: gross energy intake. ^1^ N balance (g/kg^0.75^) = (Digestible N − N in urine)/Metabolic weight; ^2^ Digestible N (g/kg^0.75^) = (N intake − N in feces)/Metabolic weight; ^3^ Digestible energy (DE) (MJ/kg^0.75^) = (GEI − Energy in feces)/Metabolic weight; ^4^ Metabolizable energy (ME) (MJ/kg^0.75^) = (DE − Energy in urine − Methane energy)/Metabolic weight; ^5^ Methane energy (MJ/kg^0.75^) = energy destined to CH_4_ formation calculated from Aguilera [19], so that it represents 10.32% of DE.

**Table 4:** Effect of olive pulp inclusion in the goat diet on ruminal short-chain fatty acids and N-NH_3_ concentration in an in vivo trial (n = 9 goats per treatment).

	Diets			
	CTL	OPD	SEM	*p*-Value
Total SCFA (µM)	61.0	57.6	2.64	0.540
Molar proportions (mol/100 mol)				
Acetate	60.9	59.9	0.840	0.554
Propionate	24.1	22.6	0.950	0.444
Isobutyrate	1.08	1.07	0.076	0.959
Butyrate	11.3	13.5	0.490	0.020
Isovalerate	0.900	0.920	0.070	0.918
Valerate	1.69	1.70	0.070	0.900
Acetate: Propionate	2.54	2.82	0.130	0.305
N-NH_3_ (mg/100 mL)	2.81	1.72	0.402	0.187

CTL: control group; OPD: group including 12% OP in the concentrate; SEM: standard error of mean; SCFA: short-chain fatty acids.

**Table 5:** Initial (day 0) and final (day 30) body weights of animals that consumed the experimental diets (control and olive pulp diet) during the on-farm trial (n = 17 and n = 24 goats for CTL and OPD, respectively).

	Diets			
Body Weight (kg)	CTL	OPD	SEM	*p*-Value
Day 0	52.0	55.2	1.14	0.168
Day 30	54.7	57.8	1.46	0.192

CTL: control group; OPD: group containing 12% OP in the concentrate; SEM: standard error of mean.

**Table 6:** Effect of the olive pulp inclusion in the diet of dairy goats on the milk yield in the on-farm trial (n = 18 and n = 20 goats for CTL and OPD, respectively).

	Diets			
	CTL	OPD	SEM	*p*-Value
Milk yield (g/d)	2160	1920	104	0.269
CF	120	109	5.15	0.318
Lactose	102	88.3	4.98	0.151
CP	80.7	78.7	3.66	0.789
Composition (%)				
CF	5.65	5.86	0.140	0.465
CP	3.79	4.17	0.090	0.036
Lactose	4.76	4.58	0.030	0.008
Total solids (CP + CF + Lactose)	14.2	14.7	0.213	0.255
FPCM (g/day)	2282	2113	0.994	0.411
Somatic cells count (×10^3^/mL)	1503	1740	359	0.746
Antioxidant activity				
Total polyphenols (mg/kg milk)	44.6	37.5	1.05	0.003
ABTS (µmol/kg milk)	1361	1301	47.6	0.540

CTL: control group; OPD: group containing 12% OP in the concentrate; SEM: standard error of mean; CF: crude fat; CP: crude protein; FPCM: fat-protein corrected milk calculated from Mancilla-Leytón et al. [25]; ABTS: 2,2′-azino-bis (3-ethylbenzothiazoline-6-sulfonic acid.

**Table 7:** Effect of the inclusion of olive pulp in the diet of dairy goats on the fatty acid composition of milk fat in the on-farm trial (n = 18 and n = 20 goats for CTL and OPD, respectively).

	Diets			
Fatty Acid (wt%)	CTL	OPD	SEM	*p*-Value
C4	1.93	1.99	0.046	0.499
C6	1.99	2.06	0.048	0.498
C8	2.32	2.35	0.042	0.772
C10	8.10	9.02	0.179	0.009
C11	0.172	0.186	0.006	0.205
C12	4.74	5.38	0.088	<0.001
C13	0.119	0.131	0.005	0.245
C14	9.53	9.72	0.147	0.528
C14:1	0.299	0.315	0.007	0.261
C15	0.699	1.01	0.047	<0.001
C15:1	0.137	0.215	0.010	<0.001
C16	29.9	28.4	0.316	0.020
C16:1	1.52	1.66	0.043	0.121
C17	0.405	0.839	0.045	<0.001
C17:1	0.247	0.448	0.027	<0.001
C18	7.88	8.09	0.200	0.622
C18:1n9t	0.679	0.693	0.025	0.779
C18:1n11t	1.21	1.30	0.031	0.136
C18:1n9c	21.5	19.6	0.347	0.004
C18:2n6t	0.219	0.189	0.010	0.145
C18:2n6c	3.08	2.79	0.083	0.079
C18:3n6 γ	0.070	0.069	0.004	0.833
C18:3n3 α	0.23.9	0.552	0.032	<0.001
C20	0.767	0.586	0.032	0.003
9c-11t CLA	0.235	0.346	0.014	<0.001
9t-11t CLA	0.066	0.112	0.007	<0.001
10t12c CLA/9c-11cCLA	0.104	0.149	0.007	<0.001
C20:1n9	0.558	0.554	0.011	0.859
C20:2	0.111	0.106	0.003	0.397
C21	0.035	0.055	0.003	0.003
C20:3n6	0.121	0.131	0.003	0.053
C20:4n6	0.271	0.228	0.003	0.035
C20:3n3	0.089	0.071	0.004	<0.001
C22	0.106	0.092	0.004	0.078
C22:1n9	0.119	0.108	0.003	0.152
C20:5n3 (EPA)	0.123	0.119	0.017	0.445
C22:2	0.044	0.061	0.003	0.001
C23	0.026	0.031	0.002	0.216
C24:0	0.002	0.009	0.000	<0.001
C24:1	0.003	0.012	0.001	<0.001
C22:5n3 (DPA)	0.188	0.166	0.006	0.060
C22:6n3 (DHA)	0.084	0.081	0.003	0.646
Summary				
SFA	68.7	69.9	0.401	0.121
MUFA	26.3	24.9	0.372	0.061
PUFA	5.04	5.18	0.085	0.418
Trans FA	2.09	2.05	0.066	0.733
MUFA/PUFA	5.27	4.84	0.100	0.033
PUFA/MUFA	0.19	0.21	0.004	0.031
CLA	0.40	0.62	0.024	<0.001
Atherogenicity index	2.22	2.34	0.044	0.198
Ratio Omega 6/omega 3	6.19	4.00	0.245	<0.001

CTL: control group; OPD: group containing 12% of OP in the concentrate; SEM: standard error of mean.; wt%: weight percentage SFA: saturated fatty acids; MUFA: monounsaturated fatty acids; PUFA: polyunsaturated fatty acids; Trans FA: trans fatty acids.

## Data Availability

All the raw data are held in the Digital CSIC public repository at the site https://doi.org/10.20350/digitalCSIC/17397.

## Abbreviations

The following abbreviations are used in this manuscript:

ABP	Agroindustrial by-products
ADF	Acid detergent fiber
ADL	Acid detergent lignin
CF	Crude fat
CLA	Conjugated linoleic acid
CP	Crude protein
CTL	Diet of control group
DDGS	Distiller’s dried grains with solubles.
DDVOP	Dried destoned virgin olive pomace
DE	Digestible energy
DHA	Docosahexaenoic acid
DIM	Days in milk
DM	Dry matter
DPA	Docosapentaenoic acid
EPA	Eicosapentaenoic acid
FAO	Food and Agriculture Organization
FPCM	Fat-protein corrected milk
GE	Gross energy
GEI	Gross energy intake
IPCC	Intergovernmental Panel on Climate Change
LCA	Life-cycle assessment
ME	Metabolizable energy
MUFA	Monounsaturated fatty acids
N	Nitrogen
NDF	Neutral detergent fiber
NSC	Non-structural carbohydrates
OBP	Olive by-products
OM	Organic matter
OP	Olive pulp
OPC	Olive pulp concentrate with 12% DM of inclusion of OP
OPD	Olive pulp diet including OPC
PUFA	Polyunsaturated fatty acids
SCFA	Short-chain fatty acids
SEM	Standard error of mean
SFA	Saturated fatty acids
VA	Vaccenic acid
